# Structural-Geometric Functionalization of the Additively Manufactured Prototype of Biomimetic Multispiked Connecting Ti-Alloy Scaffold for Entirely Noncemented Resurfacing Arthroplasty Endoprostheses

**DOI:** 10.1155/2017/5638680

**Published:** 2017-07-13

**Authors:** Ryszard Uklejewski, Mariusz Winiecki, Piotr Rogala, Adam Patalas

**Affiliations:** ^1^Department of Medical Bioengineering Fundamentals, Institute of Technology, Casimir the Great University, Chodkiewicza 30, 85-064 Bydgoszcz, Poland; ^2^Department of Process Engineering, Institute of Technology and Chemical Engineering, Poznań University of Technology, Berdychowo 4, 60-965 Poznań, Poland; ^3^Department of Spine Surgery, Oncologic Orthopaedics and Traumatology, Poznan University of Medical Sciences, 28 Czerwca 1956 135/147, 61-545 Poznań, Poland

## Abstract

The multispiked connecting scaffold (MSC-Scaffold) prototype, inspired by the biological system of anchorage of the articular cartilage in the periarticular trabecular bone by means of subchondral bone interdigitations, is the essential innovation in fixation of the bone in resurfacing arthroplasty (RA) endoprostheses. The biomimetic MSC‐Scaffold, due to its complex geometric structure, can be manufactured only using additive technology, for example, selective laser melting (SLM). The major purpose of this work is determination of constructional possibilities for the structural-geometric functionalization of SLM‐manufactured MSC‐Scaffold prototype, compensating the reduced ability—due to the SLM technological limitations—to accommodate the ingrowing bone filling the interspike space of the prototype, which is important for the prototype bioengineering design. Confocal microscopy scanning of components of the SLM‐manufactured prototype of total hip resurfacing arthroplasty (THRA) endoprosthesis with the MSC‐Scaffold was performed. It was followed by the geometric measurements of a variety of specimens designed as the fragments of the MSC-Scaffold of both THRA endoprosthesis components. The reduced ability to accommodate the ingrowing bone tissue in the SLM‐manufactured prototypes versus that in the corresponding CAD models has been quantitatively determined. Obtained results enabled to establish a way of compensatory structural‐geometric functionalization, allowing the MSC‐Scaffold adequate redesigning and manufacturing in additive SLM technology.

## 1. Introduction

The extensive development of additive technologies made it possible to design and to build the complex freeform structural constructs of metal or alloy powders. The new age, which creates new challenges and possibilities for the bioengineering design of orthopaedic implants, has begun. Today, largely owing to these technologies, it is possible to manufacture scaffolds mimicking the microstructure of natural tissue structures, to improve implant in vivo performance by enhancing their structural biocompatibility with the interfacing peri-implant bone. By using additive technologies, it is possible to manufacture precisely designed Ti-alloy microarchitectures with controlled structural and mechanical properties in the range of the properties of the bone [1, 2] and having three-dimensionally interconnective porosity with defined pore size [3, 4]. Such potential provides significant advantage for design and structural-geometric functionalization of intraosseous scaffolds for bone regeneration in vivo [5–7]. A very good example of such promising perspective for artificial joint development is the biomimetic Ti-alloy prototype of the multispiked connecting scaffold (MSC-Scaffold) for entirely noncemented and bone tissue-preserving fixation on the periarticular trabecular bone of both components of hip resurfacing arthroplasty (RA) endoprostheses. This biomimetic fixation technique was invented by Rogala [8–10], designed and manufactured using Selective Laser Melting (SLM) technology, and developed by our research team [11–14]. The spike system of our MSC-Scaffold prototype mimics the interdigitation anchorage system of the articular subchondral bone. During implantation into the trabecular bone marrow lacunae, the spikes will cause the controlled destruction of bone trabeculae at the desired osteoinductive level, allowing the promotion of bone tissue ingrowth into the MSC-Scaffold's interspike space.

Our MSC-Scaffold prototype, manufactured owing to the additive technology, opens a new generation of RA endoprostheses, prospective with their first at all biomimetic fixations in the bone of articular components, which can be applied for most diarthrodial joint arthroplasties (hip, knee, shoulder, elbow, and so forth) used in orthopaedic surgery treatment. This new generation of RA endoprostheses is prominent with the biomimetism of MSC-Scaffold respecting the microstructure of the periarticular subchondral and cancellous bone tissue, as well as with the close-to-natural load transfer, which—as in biomechanical environment of the natural hip joint—goes through the bone trabeculae in the head and the neck of the femur and then along the femoral shaft.

Assessment of dimensional differences between the designed CAD models and the SLM-manufactured MSC-Scaffold prototypes provides key information about the necessary modifications in design of the CAD model of the MSC-Scaffold prototype taking into account the adjustments of the technological constraints of chosen technology. The major purpose of this work is determination of constructional possibilities for the compensatory structural-geometric functionalization of the manufactured SLM technology prototype of the biomimetic multispiked connecting scaffold (MSC-Scaffold), necessary for the bioengineering design and SLM manufacturing of the subsequent prototypes of partial and total RA knee and hip endoprostheses with the MSC-Scaffold.

## 2. The Structural-Geometric Pro-Osteoconduction Potential: Theoretical Background

The pro-osteoconduction potential of implant porous coatings and scaffolds is conditioned by the microgeometric properties of their pores and also by the chemical composition of bone-contacting surfaces. The osteoconductive behaviour of implant porous coating and/or scaffolds related to their morphological features can be considered as their structural-geometric pro-osteoconductive potential. Such potential determines the required microgeometric pore compartments of the interconnected pore space of implant porous coatings and scaffolds, allowing the ingrowing bone tissue to create its fundamental structural units of the microvascular structural tissue level, like osteons or trabeculae, respectively, in the cortical bone and trabecular bone tissue.

In case of bone ingrowth into the porous coatings and the porous scaffolds, it has to be underlined that the morphological or the topological factors (pore size, porosity, and pore structure) are the most important factors influencing bone ingrowth [15], and this structural-geometric pro-osteoconductive potential can be controlled by additive manufacturing [16, 17]. 3D pores as the microenvironment of bone ingrowth play an important role in osteoconductivity and bone formation. Pore size is a vital parameter that influences implant-induced osteogenesis, as it provides room for the migration and proliferation of osteoblasts (i.e., osteoconduction) [15, 18]. The ideal pore structure should allow nutrient supplementation and cell adhesion to the scaffold [19]. Too small a pore size may hinder the transportation of oxygen and nutrients to the center of the scaffold, inhibiting cell proliferation and maturation, and resulting in poor bone-implant bonding, while too large a pore size is associated with a rather low bone ingrowth ratio [15]. Good interconnectivity and well-controlled pore throat size is essential for vascular tissue ingrowth and bone conductivity (i.e., osteoconduction) [20, 21]. Albrektsson and Johansson [22], while studying the osteoconduction and remodelling in vivo, have come to the conclusion that the full vascularization is necessary for proper bone formation. Providing enough room to accommodate the ingrowing bone tissue means to assure the microstructural conditions for the revascularization and full bone mineralization—both in case of pore space of porous coatings in general and in case of the interspike space of the MSC-Scaffold prototype in particular. The structural-geometric pro-osteoconduction potential of the MSC-Scaffold is therefore the most important factor influencing the bone ingrowth and affecting the peri-implant bone tissue regeneration to provide the successive bone-implant fixation.

The structural-geometric pro-osteoconduction potential of the porous coating outer layer can be described by the poroaccessibility parameter set and determined using the methodology that worked out in our research team [23–26]. The concept of the poroaccessibility together with all definitions and the mathematical formulas for calculation of the poroaccessibility parameters are given in [26]. To determine this potential in case of the MSC-Scaffold prototype, the analogous structural-geometric analyses of its interspike space have to be performed, regarding the expected requirements of its structural-geometric pro-osteoconduction potential.

Our previous findings, dealing with implant porous coatings [27, 28], showed that the poroaccessibility parameter set characterizes major aspects of the morphological features of the outer layer of porous coatings, including those describing the spatial, volumetric, hybrid, and functional properties of the implant surface. In Table 1, the set of parameters for characterization of the poroaccessibility of the intraosseous implant porous coating outer layers [23–26] is presented, and the proposal for the equivalent parameters recommended for determination of the structural-geometric pro-osteoconduction potential of the MSC-Scaffold prototype design is given. Analogous to the term poroaccessibility, the structural accessibility of the MSC-Scaffold prototype describes the morphological aspect of its pro-osteoconduction potential of its interspike space.

*The effective height H*_ef_ of spikes is considered as the spike's height above the surface of the spherical cap. It should be measured as length of the line segment-overlapping axis of the spike from its top to the point intersecting the inner or outer arc representing the meridian of the spherical cap of femoral and acetabular components of the THRA endoprosthesis, respectively.

*The representative interspike distance D*_is-rep_ should be taken as the arithmetic mean of distance between the spikes taken from established levels of the MSC-Scaffold prototype spikes' height. In case of both components of the THRA endoprosthesis, the spikes are designed in a parametrically ordered arrangement and located in concentric parallels of latitude with specified distance, both circumferentially and radially.

*The relative surface fraction of the interspike compartment cross-section ϕ*_Sis-rep_ of the MSC Scaffold prototype is the function of *the representative interspike distance D*_is-rep_. It is defined as the ratio of area of cross-section of the interspike compartment and total area of cross-section of the examined fragment of the MSC-Scaffold prototype. It should be estimated as the arithmetic mean of particular ratios taken from the established levels of the height of the MSC-Scaffold prototype spikes.

The three-dimensional parameters describing the volume of the geometric shape of the interspike space, both absolutely, and in relation to other volumetric or areal quantity, can be generally estimated as the product of the area of its base and height. Since, the spikes of the MSC-Scaffold prototype are assumed to be designed in the parametrically ordered arrangement, the volume of interspike compartment of the examined fragment of the MSC-Scaffold prototype available for ingrowth of the trabecular bone can be approximately evaluated on the basis of known values of *the representative interspike distance D*_is-rep_ and measured value of *the effective height H*_ef_. Thus, *the relative volume fraction of the interspike space ϕ*_Vis-ef_ can be estimated as the ratio of volume of the interspike compartment of the examined fragment of the MSC-Scaffold prototype and the total volume of the examined fragment of the MSC-Scaffold prototype, whereas *the index of capacity of the interspike compartment V*_is_ enables the potential volume available for ingrowth of the trabecular bone into the interspike compartment of the MSC-Scaffold per surface unit.

*The representative angle of the interspike space osteoaccessibility Ω*_rep-is_ is defined as the inclination angle of the lateral surface of the MSC-Scaffold spikes. It can be calculated according to the formula *Ω*_rep−is_ = 90° − *α*_i_/2, where *α*_i_ is the vertical angle of spikes.

In CAD models, the spikes of the MSC-Scaffold prototype are designed as regular pyramids with a square base. *The nominal height H*_n_ of the pyramid is considered to be the height measured in the CAD model from its base to the apex. The *H*_n_/*R ratio*—the ratio of *the nominal height H*_n_ of the pyramid to the radius of the circumcircle of the pyramid's base—should be, according to Rogala's patent assumptions [8–10], at least 5. Because of the high value of *H*_n_/*R ratio* in spikes of the MSC-Scaffold, the values of vertical angle *α*_i_ are relatively small. Therefore, the influence of *the representative angle of the interspike space osteoaccessibility Ω*_rep-is_ has practically negligible influence for the volumetric parameters determining the poroaccessibility of the MSC-Scaffold prototype.

*The bone-implant interface adhesive surface enlargement index ψ*_is_ is the ratio of the lateral surface area of the MSC-Scaffold spikes to the total area of the spikes' location (including the surface between spikes). Increase in *the effective height H*_ef_ of SLM-manufactured spikes of the MSC-Scaffold directly translates into the enlargement of its adhesive properties. In accordance with the conclusions presented in work [28] and Rogala's patent assumptions [8–10], the parameter assessing the adhesive surface enlargement of the MSC-Scaffold is crucial for determination of the structural conditions for bone-implant interactions. Its linear dependence *to the effective height H*_ef_ of the MSC-Scaffold spikes has been revealed in [29].

Concluding the above, and also taking into account findings regarding the interrelations between the poroaccessibility parameters describing the porous coating outer layers indicated in papers [27, 28], enables us to assume that, in the case of the MSC-Scaffold, the parameters proposed as their equivalents can be used interchangeably as adequate. Therefore, the “poroaccessibility” of the MSC-Scaffold and, consequently, the structural-geometric pro-osteoconduction potential of the MSC-Scaffold is dependent on *the effective height H*_ef_ of spikes and *the representative interspike distance D*_is-rep_.

Since the patent requirements restrict the constancy of *the representative interspike distance D*_is-rep_, in this paper, the enhancement of *the structural-geometric pro-osteoconduction potential of the of MSC-Scaffold* interspike space is analyzed, as achieved by the change in *the effective height H*_ef_ of the MSC-Scaffold spikes. However, the necessity of slight modification of the distance between the spikes' bases in the MSC-Scaffold design has been concluded in our previous research carried out in animal model and in osteoblast culture [30]; the limited correctability (with a scale of hundreds of micrometres), which is restricted by the biological factors [31], makes this change insignificant in comparison to that obtained by the change in *the effective height H*_ef_ of the MSC-Scaffold spikes (with a scale of millimetres).

## 3. Materials and Methods

The CAD models of femoral and acetabular components of our THRA endoprosthesis prototype [14] were designed as solids of revolution generated by revolving the specific contour in the three-dimensional space about the axis coplanar with contour. Both the endoprosthesis components are in the shape of spherical caps. In the case of the femoral component, the MSC-Scaffold prototype is designed on the inner surface of the spherical cap, while in the case of the acetabular component, it is designed on the outer surface of the spherical cap.

The multilateral spikes of the MSC-Scaffold prototype are arranged in parallels of latitude—there are 20 for the femoral component and 17 for the acetabular component. The length of the square side in the pyramid's base was established as 0.5 mm in the CAD model. The bases of all pyramids are located under the surface of the spherical cap, so that the two vertexes of their side, located more distant from the spherical cap central axis, lie at the surface of the spherical cap. The curves created at the junction of intersecting surfaces of the spherical cap and particular pyramids create the bases of the particular MSC-Scaffold prototype spikes. The edges of bases of the adjacent spikes were originally designed in contact with each other, both circumferentially and radially.

The three geometric variants of spikes, which can be distinguished in the femoral component CAD model, are arranged in particular concentric parallels of latitude: spikes in the first 12 parallels of latitude (counting from the central spike) have the designed *H*_n_/*R ratio* equal to 8; then, spikes located in the next 5 parallels of latitude have the designed *H*_n_/*R ratio* equal to 9; and finally, the spikes located in the last 3 parallels of latitude, close to the equator, have the designed *H*_n_/*R ratio* equal to 10. All of the spikes located in concentric parallels of latitude of the acetabular component have the designed *H*_n_/*R ratio* equal to 10. *The nominal heights H*_n_ of the MSC-Scaffold prototype spikes are 2.828 mm, 3.182 mm, and 3.536 mm, respectively, for the *H*_n_/*R ratios* equal to 8, 9, and 10.

The assumed effect of theoretical enhancement of the structural pro-osteoconduction potential of the MSC-Scaffold prototype designed in CAD model was evaluated in comparison to the corresponding SLM-manufactured prototypes of the MSC-Scaffold.

The SLM-manufactured prototype of the THRA endoprosthesis [14] was examined with the use of confocal microscopy. The essential examination was performed on a variety of specific SLM-manufactured specimens, representing fragments of the MSC-Scaffold prototype of both THRA endoprosthesis components by the digital measurement of *the effective height H*_ef_ of the MSC-Scaffold prototype spikes.

**Figure 1:**
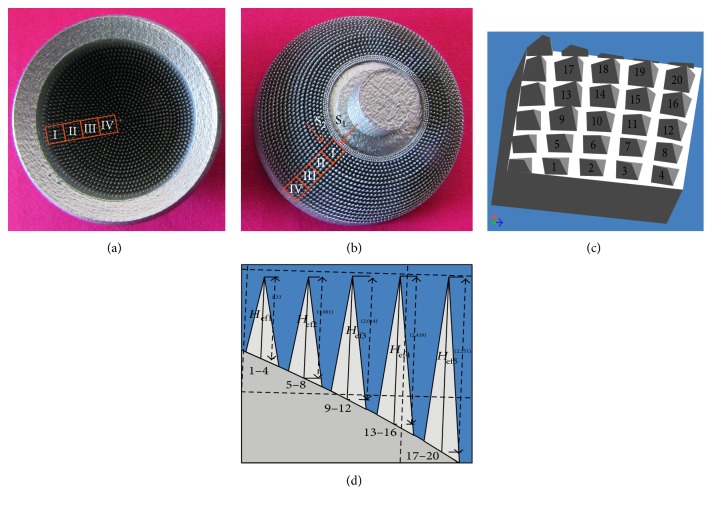
The femoral (a) and the acetabular (b) components of the SLM-manufactured prototype of the THRA endoprosthesis with the MSC-Scaffold. The square frames mark scanned regions of the MSC-Scaffold prototype; the 3D CAD file of the MSC-Scaffold fragment (c) corresponding to the exemplary scanned region on the acetabular component of the prototype of the THRA endoprosthesis, marked with Roman numeral I. The perspective view is marked by the arrow and letter “S_1_”; spikes of the MSC-Scaffold (d). The side view is marked by arrow and letter “S_2_”.

To compare the prototype of the MSC-Scaffold with its theoretical CAD model, the 3D confocal scanning and profile measurements of the scaffold prototype were performed with the use of the Olympus Lext OLS4000 microscope equipped with the MPLFLN×5 objective. Scanning was performed on the neighbouring regions (size: 2560 *μ*m × 2560 *μ*m) of the MSC-Scaffold prototype localized along the radius of both components (femoral and acetabular) of the THRA endoprosthesis prototype. In Figures 1(a) and 1(b), the femoral and the acetabular components of the THRA endoprosthesis prototype are presented, and the measured regions are marked with the square frames. The 2D and 3D representations of each scanned region were visualized in a software associated with the confocal microscope. The profile lines were plotted radially through the spikes' peaks to measure *the effective height H*_ef_ of the spikes and *the radius R_x_* of the spike bases. The approximate values of the *H*_ef_/*R_x_ ratios* were calculated to refer to values of the *H*_n_*/R ratios* assumed in the CAD model. In Figure 1(c), the fragment of the 3D CAD file corresponding to the selected region is shown along with the labelled spikes of the MSC-Scaffold prototype. Since, spikes of the MSC-Scaffold prototype are arranged in concentric parallels of latitude, in Figure 1(d), the measurement sequence of the representative spikes from 5 internal parallels of latitude is shown.

In CAD models of the specimens representing fragments of the MSC-Scaffold, the shapes of contour in the half sections of the THRA endoprosthesis components were extruded to form the three-dimensional solids similar to the spherical cap sector. Spikes of the MSC-Scaffold prototype were set in lines along the arc, representing meridian of the spherical cap sector. In each of the two designed series of the MSC-Scaffold fragments, an attempt of enhancement of the structural pro-osteoconduction potential of the MSC-Scaffold has been assumed by the specific modification of geometric constructional properties of the spikes. In the first series, the potential was theoretically enhanced in CAD models by the change in *the nominal height H*_n_ of spikes, while in the second series, *the nominal height H*_n_ of spikes was not changed, but the geometric shape of spikes was modified by truncating the pyramid and changing the apex angle of it.

The first specimen in both series representing the MSC-Scaffold prototype fragments, treated as the basis for others, was labelled *FCS_I* for the femoral component specimen and *ACS_I* for the acetabular component specimen. It was reproduced on the basis of the geometric variant of the prototype of THRA endoprosthesis presented in paper [14]. In CAD models of the first series of 9 MSC-Scaffold prototype variants representing the femoral THRA component (labelled *FCS_I*–*FCS_IX*), the assumed *H*_n_/*R ratio* of spikes were designed as increasing from 8, 9, and 10 up to 16, 17, and 18, respectively, for 3 specific variants of spikes. In CAD models of the first series of 9 MSC-Scaffold prototype variants representing the acetabular THRA component (labelled *ACS_I*–*ACS_IX*), the assumed *H*_n_/*R ratio* of spikes was designed as increasing from 10 up to 18. The second series of 9 MSC-Scaffold prototype variants was designed on the basis of the following MSC-Scaffold prototype variants selected from the first series: *FCS_I*, *FCS_V*, *FCS_VIII*, *ACS_I*, *ACS_V*, and *ACS_VIII*. In CAD models of these specimens, spikes were designed in the shape of a truncated pyramid, with a square on the top. The length of the square side was equal to 0.1 mm, 0.2, and 0.3 mm for each variant selected as the point of departure.

Verification of the enhancement of structural pro-osteoconduction potential of the MSC-Scaffold prototype assumed in particular CAD models was examined in the corresponding SLM-manufactured specimens. Four sets of specimens were manufactured at once in the Renishaw AM250 machine (Renishaw plc, UK) of Ti-6Al-4V powder. The process parameters applied during the SLM manufacturing of the prototypes were laser—150–200 watt, layer thickness—30 *μ*m, laser spot size—0.07 mm, scan speed—0.35–5 m/s, and laser energy density—80–160 J/mm^3^.

**Figure 2:**
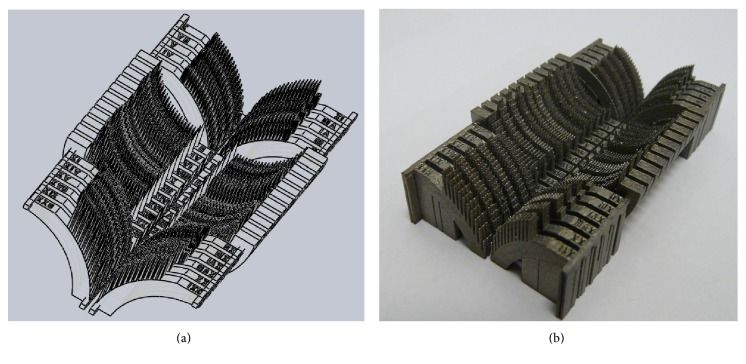
The CAD models (a) and just SLM-manufactured set (b) of the specimens representing fragments of the THRA endoprosthesis components with various geometric variants of the MSC-Scaffold.

In Figure 2(a), the CAD model of the specimen set representing fragments of the femoral and the acetabular components of the THRA endoprosthesis with various geometric variants of the MSC-Scaffold is presented, while the set of SLM-manufactured specimens is shown in Figure 2(b).

After manufacturing, the sets of specimens were separated and segregated. Each specimen was digitally photographed in high resolution with the scale bar with the use of the digital photo camera DSC-H1 (Sony, Japan). *The effective height H*_ef_ of each spike of the SLM-manufactured MSC-Scaffold fragments was measured manually six times in a PC-based system using the professional software tool ImageJ (NIH). Before measurements, the software tool was precisely calibrated with the use of the scale bar. *The effective height H*_ef_ of spikes in CAD models was measured using the built-in measurement tool in the Autodesk Inventor Professional 9 CAD software.

## 4. Results

**Figure 3:**
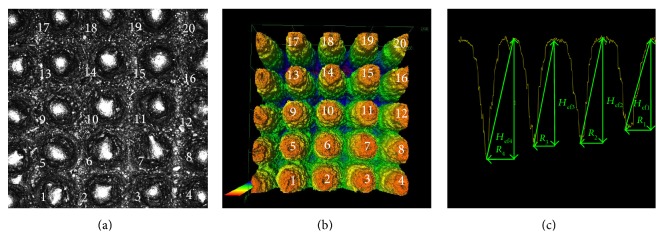
The 2D view (a) and the 3D representation (b) of the exemplary region of the MSC-Scaffold prototype scanned with the confocal microscope (this corresponds with the sector marked with Roman numeral I on the acetabular component of prototype of the THRA endoprosthesis, see Figure 1(b)); the exemplary profile through the spikes' peaks at which the effective height *H*_ef_ of spikes and the radius *R_x_* of the spikes' bases were measured (c).

In Figures 3(a) and 3(b), the 2D photograph and the 3D perspective view of the exemplary region of the MSC-Scaffold prototype are presented. This region corresponds with the fragment of the 3D CAD file presented in Figure 1(c). In Figure 3(c), the manner of measurement of *the radius R_x_* of the spikes' bases and *the effective height H*_ef_ of the four exemplary spikes of the MSC-Scaffold prototype are shown, measured alongside the profile running through the peaks of the spike and the deepest points between the spikes.

**Figure 4:**
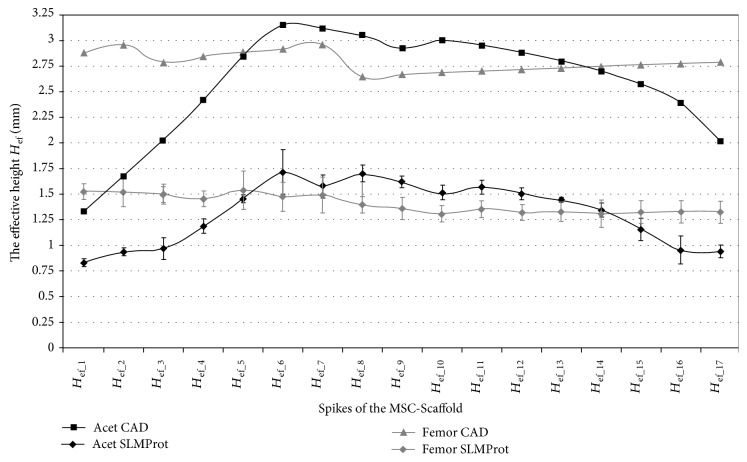
The effective height *H*_ef_ of the MSC-Scaffold's spikes measured in CAD model in comparison to the effective height *H*_ef_ of the MSC-Scaffold's spikes measured in the SLM-manufactured components (femoral and acetabular) of the prototype of THRA endoprosthesis.

**Figure 5:**
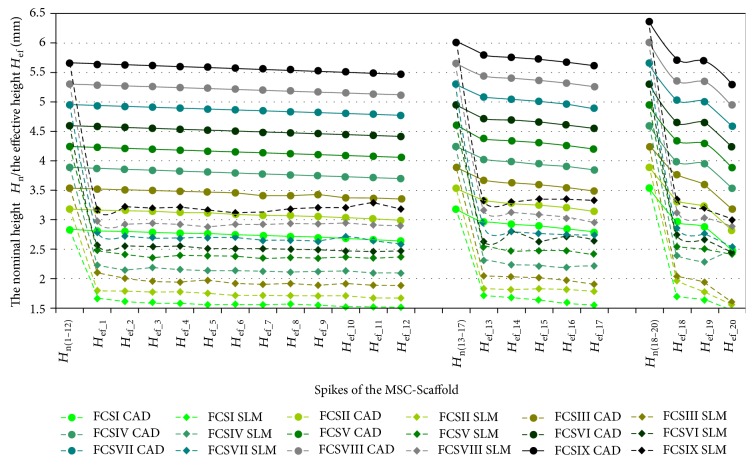
The effective height *H*_ef_ of the MSC-Scaffold's spikes measured in the CAD models in comparison to the effective height *H*_ef_ of the MSC-Scaffold's spikes measured in the SLM-manufactured specimens representing fragments of the MSC-Scaffold prototype of the femoral (*FCS_I*-*FCS_IX*) component of the THRA endoprosthesis.

**Figure 6:**
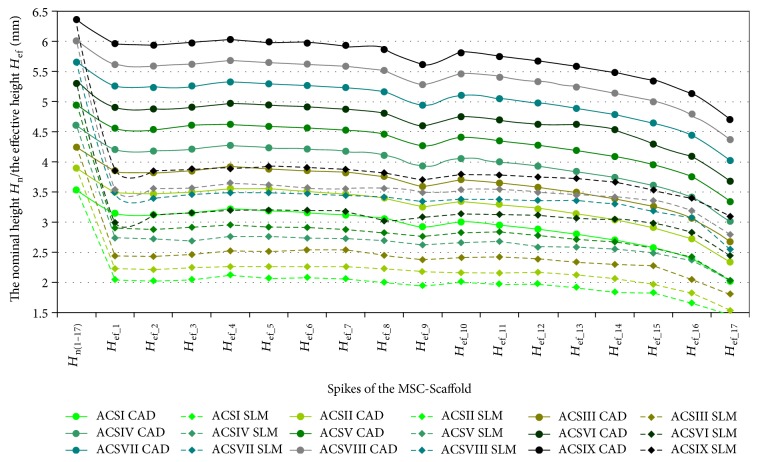
The effective height *H*_ef_ of the MSC-Scaffold's spikes measured in the CAD models in comparison to the effective height *H*_ef_ of the MSC-Scaffold's spikes measured in the SLM-manufactured specimens representing fragments of the MSC-Scaffold prototype of the acetabular (*ACS_I*-*ACS_IX*) component of the THRA endoprosthesis.

In Figures 4, 5, and 6, the results of measurements of *the effective height H*_ef_ of spikes of the MSC-Scaffold prototype are presented in particular CAD models versus the corresponding SLM-manufactured specimens.  Figure 4 refers to the components of the THRA endoprosthesis prototype [14]. Figures 5 and 6 refer to the first series of geometric variants of the MSC-Scaffold prototype. The measured values of *the effective height H*_ef_ of spikes are distributed as a function of its location along the arc, representing meridian of the spherical cap of femoral and acetabular components of the THRA endoprosthesis and are labelled with the number of particular concentric parallels of latitude of its location. The measurement results are presented in reference to *the nominal height H*_n_ of the pyramid used in CAD model as the basis and are labelled *H*_n(1–12)_, *H*_n__(13–17)_, and *H*_n(18–20)_ in the case of the femoral component and *H*_n(1–17)_ in the case of the acetabular component of the THRA endoprosthesis. Results of all measurements were averaged and are presented as mean values (±standard deviations). For particular sets of curves, the error bars were omitted for better legibility of the diagrams.

**Figure 7:**
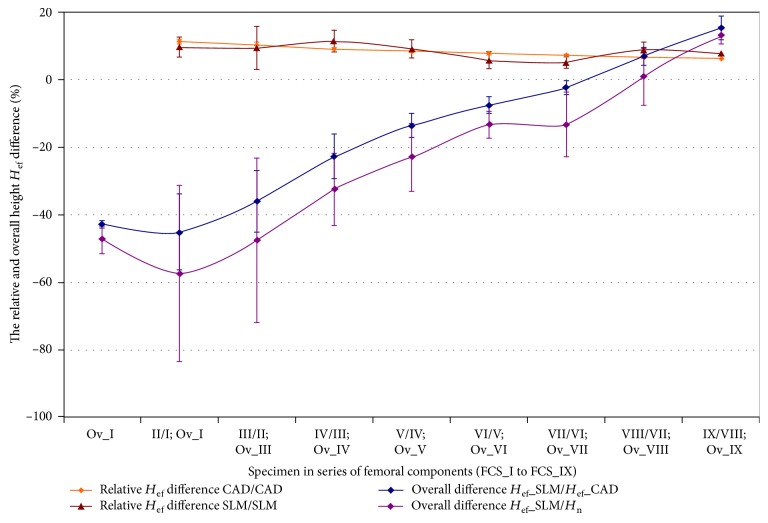
Curves of the relative increase of the effective height *H*_ef_ of the MSC-Scaffold's spikes in the consecutive specimens in a series of 9 specimens representing the femoral component—*FCS_I* to *FCS_IX* together with the curves showing the overall difference in the effective height *H*_ef_ in the particular SLM manufactured specimens. The curves are presented in relation to the effective height *H*_ef_ in the CAD model of the MSC-Scaffold prototype treated as the basis (*FCS_I* for femoral components) and in relation to the nominal height *H*_n_ of the MSC-Scaffold's spikes.

**Figure 8:**
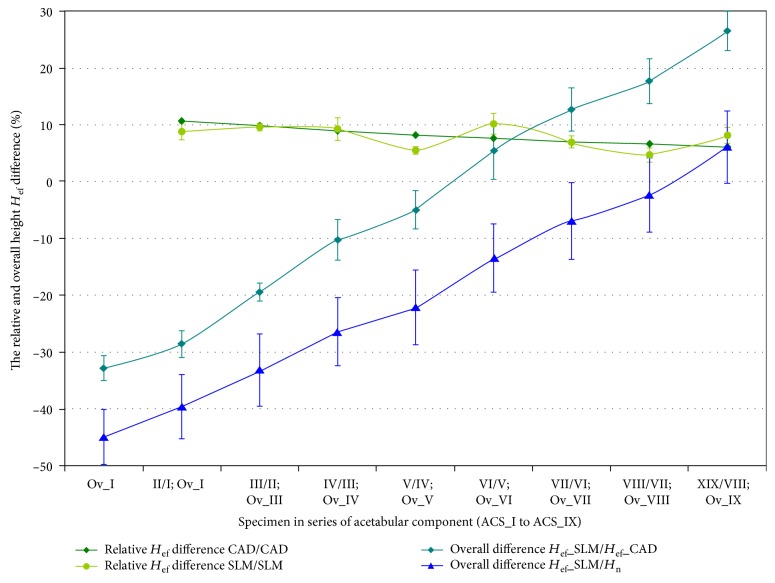
Curves of the relative increase of the effective height *H*_ef_ of the MSC-Scaffold's spikes in the consecutive specimens in a series of 9 specimens representing the acetabular component—*ACS_I* to *ACS_IX*.

The specific modification of geometric constructional properties of the MSC-Scaffold's spikes in the first series of specimens has assumed the stepwise increase of the *H*_n_/*R ratio* of spikes. In Figures 7 and 8, curves of the relative increase of *the effective height H*_ef_ of the MSC-Scaffold's spikes in the consecutive specimens are presented in a series of 9 specimens representing the femoral component (Figure 7) and the acetabular component (Figure 8) of the THRA endoprosthesis. These curves are presented together with curves showing the overall difference in *the effective height H*_ef_ in the particular SLM-manufactured specimens in relation to *the effective height H*_ef_ in the CAD model of the MSC-Scaffold prototype treated as the basis and in relation to *the nominal height H*_n_ of the MSC-Scaffold's spikes.

**Figure 9:**
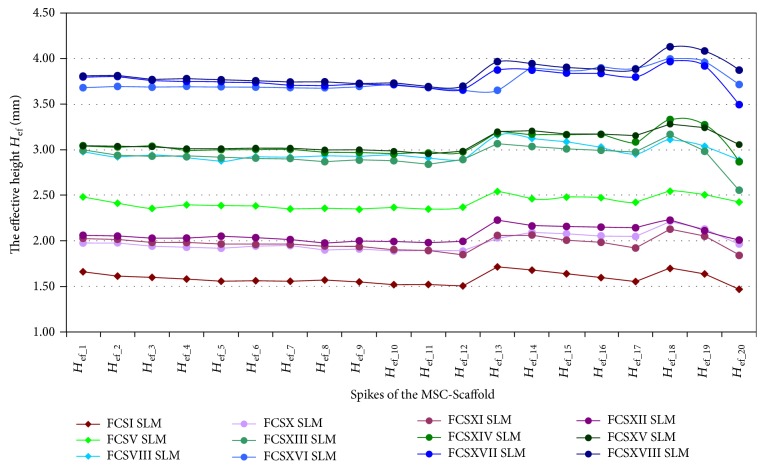
The effective height *H*_ef_ of the MSC-Scaffold's spikes measured in the second series of 9 variants of the SLM-manufactured specimens of the MSC-Scaffold prototype representing the femoral component fragments (labelled *FCS_X*–*FCS_XVIII*) of the THRA endoprosthesis in relation to the effective height *H*_ef_ measured in the SLM-manufactured specimen variants used as the basis for each particular subgroup: FSC_I, FSC_V, and FSC_VIII.

**Figure 10:**
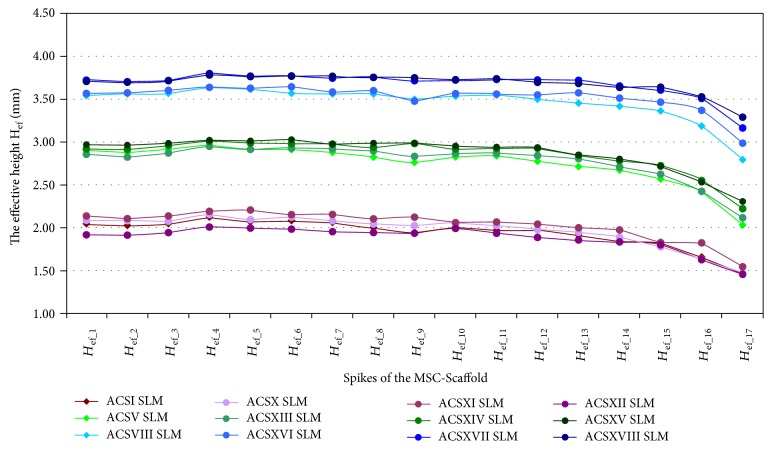
The effective height *H*_ef_ of the MSC-Scaffold's spikes measured in the second series of 9 variants of the SLM-manufactured specimens of the MSC-Scaffold variants representing the acetabular fragments (labelled *ACS_X*–*ACS_XVIII*) of the THRA endoprosthesis in relation to the effective height *H*_ef_ measured in the SLM-manufactured specimen variants used as the basis for each particular subgroup: ASC_I, ASC_V, and ASC_VIII.

In Figures 9 and 10, the results of the measurement of *the effective height H*_ef_ of spikes of the MSC-Scaffold prototype in the second series of 9 MSC-Scaffold prototype variants are presented. These sets of results are presented in relation to the results of measurements of *the effective height H*_ef_ in the SLM-manufactured specimen variants used as the basis for each particular series.

## 5. Discussion

The values of *the effective height H*_ef_ of spikes of the MSC-Scaffold prototype in the SLM-manufactured prototype of the THRA endoprosthesis (Figure 4) are significantly lower (by 48 ± 9% for the femoral component and by 51 ± 9% for the acetabular component) in relation to their equivalents in the corresponding CAD models. This significantly reduces the structural pro-osteoconduction potential of the MSC-Scaffold prototype assumed in the CAD model and consequently decreases the values of the resulting *H*_ef_/*R ratios*. Even if those ratios were assumed to be 8–10 in the CAD models, their values in the SLM-manufactured prototypes were crucially below 5.

*The effective height H*_ef_ of all spikes measured in the CAD files is lower than its *nominal height H*_n_ (Figures 5 and 6). This difference arises from the fact that the bases of all spikes are located under the surface of the spherical cup. The relative differences between *the effective height H*_ef_ and *the nominal height H*_n_ of the MSC-Scaffold's spikes vary with their locations along the arc representing meridian of the spherical cap and are significant in the case of the spikes nearest to the equator of the specific spherical cap. The differences range from 0.40 ± 0.10% to 5.0 ± 1.0% in the case of the first 12 spikes, from 5.0 ± 1.0% to 9.0 ± 2.0% in next 5 spikes, and from 12 ± 2% to 23 ± 5% for 3 terminal spikes located along the outer arc, representing meridian of the femoral THRA endoprosthesis component. In the case of the acetabular component, the differences are from 8.0 ± 2.0% up to 33 ± 6%.

It reduces the structural pro-osteoconduction potential of the MSC-Scaffold prototype and it becomes meaningful in the region of terminal spikes of the MSC-Scaffold prototype, designed close to the equator of spherical cap, where the arch curvature is quite high. To provide the uniformity of the structural pro-osteoconduction potential in the entire interspike space of the MSC-Scaffold prototype of the THRA endoprosthesis, the compensation of differentiation of *the effective height H*_ef_ of the adequate spikes located along the arc representing meridian should be considered.

The difference between *the effective height H*_ef_ values of the MSC-Scaffold's spikes measured in the CAD models versus the SLM-manufactured specimens was estimated at the level of 43 ± 2% and 44 ± 1%, respectively. The analysis has shown substantial difference between the designed CAD models of the MSC-Scaffold and the prototypes manufactured on its basis in the SLM technology. This result corresponds with the preliminary examination of the additively manufactured prototypes of both components of the THRA endoprosthesis performed with confocal microscopy.

The assumed stepwise increase in the spike *H*_n_/*R ratio*, which translates into the linear increase in *the nominal height H*_n_, has resulted in the linear increase of *the effective height H*_ef_ in CAD models of both series of specimens (Figures 7 and 8). The relative increase of *H*_ef_ measured in the SLM-manufactured specimens oscillates around the same values as those of the CAD models. The rate of growth diminishes from 11.3 ± 0.5% to 6.3 ± 0.2% and 11.0 ± 1.0% to 6.0 ± 1.0% in case of specimens representing the femoral and the acetabular component of the THRA endoprosthesis, respectively. The overall relative increase in *H*_ef_ in these series of specimens is 50 ± 2% and 49 ± 3% for the femoral component fragments and the acetabular component fragments, respectively.

The curves showing the overall difference of *the effective height H*_ef_ measured in the successive SLM-manufactured specimens of both series in reference to equivalents measured in particular CAD models of the first specimen in adequate series of the MSC-Scaffold fragments show that the enlargement of the *H*_n_/*R ratio* by at least 7 and 6, for the femoral and acetabular component, respectively, is required to manufacture the MSC-Scaffold with spikes having an *effective height H*_ef_ as primarily designed in the CAD model. Obtaining the structural pro-osteoconduction potential of the MSC-Scaffold prototype, as assumed by the design of the specific *H*_n_/*R ratio*, involves the increase in *the effective height H*_ef_ by at least 7 and 9 in the cases of the femoral and acetabular component, respectively.

Modification of the geometric shape of spikes by truncating the pyramid and changing the apex angle of the pyramid has resulted in the increase of *the effective height H*_ef_ of the MSC-Scaffold spikes in the SLM-manufactured specimens (Figure 9) by 20 ± 2% to 22 ± 2% in relation to the SLM-manufactured specimens used as the basis for each subgroup. The very slight increase, in the order of 1%, can be observed for *H*_ef_ in the consecutive specimens within each subgroup.

Considerably, smaller increase was observed for the specimens representing fragments of the MSC-Scaffold of the acetabular components of the THRA endoprostheses (Figure 10). Even though the increase for particular spikes reached values of up to 10% in relation to the SLM-manufactured specimens used as the reference for each subgroup, the large discrepancy between the data, especially in case of spikes of the MSC-Scaffold located in the terminal parallels of latitude, closest to the equator of the specific spherical cap, produces relatively high values of the standard deviations of the mean values.

**Figure 11:**
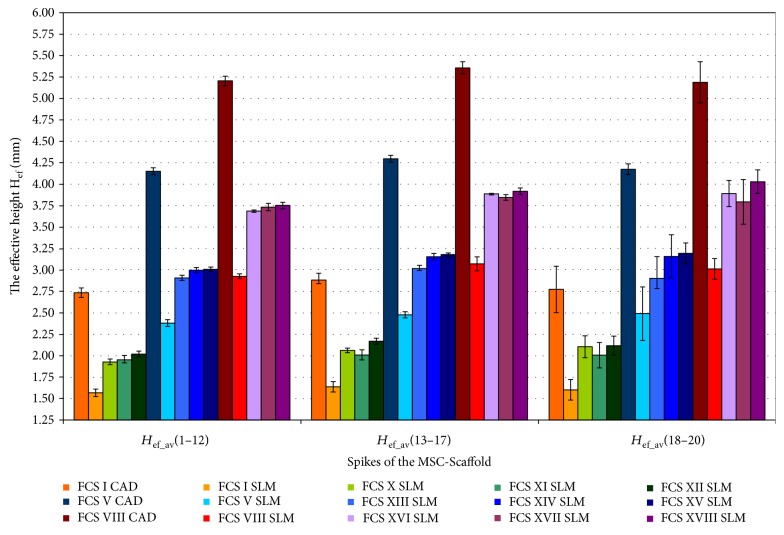
The overall effect of the possible enhancement of the interspike structural-geometric pro-osteoconduction potential of the MSC-Scaffold (numbers 1–12, 13–17, and 18–20 represent the particular concentric parallels of latitude of the spikes' location).

To summarise, the overall effect of the possible enhancement of the structural pro-osteoconduction potential of the MSC-Scaffold is presented in Figure 11 on the example of three variants of the MSC-Scaffold spikes designed in the THRA endoprosthesis femoral component. *The nominal height H*_n_ of spikes, as designed in CAD model, was 2.828 mm, 3.182 mm, and 3.536 mm (cf. Figure 10):

- Spikes of the MSC-Scaffold located in the CAD model along the arc representing the meridian of the spherical cap have *H*_ef_ values equal to 2.8 ± 0.1 mm and are lower by 3.3%, 9.4%, and 26.8% in reference to *the nominal height H*_n_.
- *The effective height H*_ef_ of spikes of the MSC-Scaffold in SLM-manufactured specimens is 1.6 ± 0.1 mm and is about 42% lower in relation to the CAD files.
- For the first variant of structural pro-osteoconduction potential enhancement, only the 8 SLM-manufactured specimens of the series had the higher value of *the effective height H*_ef_.
- For the second variant of the structural pro-osteoconduction potential enhancement, the increase in *H*_ef_ value is possible by about 20% in relation to the SLM-manufactured specimens used as the basis for each subgroup.
- The most favourable enhancement of the structural pro-osteoconduction potential of the MSC-Scaffold can be obtained by the combined modification of the design of the MSC-Scaffold spikes' in the CAD model. For example, the extension of *the nominal height H*_n_ resulting in growth of the *H*_n_/*R ratio* by 4 and the simultaneous truncating of pyramids in the CAD model to obtain the square on its top with the length of the square side equalling 0.1 mm to 0.3 mm allows achieving the value of *the effective height H*_ef_ in the SLM-manufactured prototype at the same level as designed in the CAD model of the prototype [14].

## 6. Conclusions

The examination performed on a variety of specimens representing fragments of the MSC-Scaffold prototype of the both THRA endoprosthesis components (femoral and acetabular) using confocal microscopy scanning following geometric feature measurements has allowed evaluating the constructional possibilities to affect its structural pro-osteoconduction potential, defined and determined in this paper. The reduced structural pro-osteoconduction potential in the SLM-manufactured Ti-alloy prototypes versus corresponding CAD models has been stated, and the technological constraints of selective laser melting formation of the MSC-Scaffold prototype were quantitatively determined. The obtained results have allowed us to revise the constructional assumptions of the primary prototype of the MSC-Scaffold [14] and have provided the key information about the necessity of taking into account the found technological constraints of SLM technology in establishing constructional directives, that is, a way of compensatory structural functionalization, for adequate engineering design of subsequent prototypes of partial and total RA endoprostheses of the knee and hip joints with MSC-Scaffold.

## Figures and Tables

**Table 1:** The set of stereometric parameters for characterization of the poroaccessibility of intraosseous implant porous coating outer layers and the equivalent parameters proposed for determination of the interspike structural-osteoconductive potential in the MSC-Scaffold prototype.

The poroaccessibility of the intraosseous implant porous coating outer layer can be evaluated by the following parameter set	The MSC-Scaffold prototype structural accessibility for ingrowing bone tissue can be assessed by the proposed parameters set
The effective pore depth *p*_def_	The effective height *H*_ef_
The representative pore size *p*_Srep_	The representative interspike distance *D*_is-rep_
The effective volumetric porosity *ϕ*_Vef_	The relative volume fraction of the interspike space *ϕ*_Vis-ef_ *= f*(*H*_ef_, *D*_is-rep_)
The representative surface porosity *ϕ*_Srep_	The relative surface fraction of the interspike compartment cross-section *ϕ*_Sis-rep_ *= f*(*D*_is-rep_)
The index of the porous coating outer layer space capacity *V*_PM_	The index of the capacity of the interspike space *V*_is_ = *f*(*H*_ef_, *D*_is-rep_) [mm^3^/cm^2^]
The representative angle of the poroaccessibility *Ω*_rep_	The representative angle of the interspike space osteoaccessibility *Ω*_rep-is_ = 90°−*α*_i_/2, *where α*_i_* is* the vertical angle of spikes
The bone-implant interface adhesive surface enlargement index *ψ*	The bone-implant interface adhesive surface enlargement index *ψ*_is_ *= f*(*H*_ef_, *Ω*_rep-is_)
